# Factors associated with elevated risk of depression among Brazilian truck drivers: a cross-sectional study

**DOI:** 10.1192/j.eurpsy.2026.11437

**Published:** 2026-07-31

**Authors:** E. B. Camargo Júnior, R. C. D. D. Silva

**Affiliations:** University of Rio Verde, Rio Verde, Brazil

## Abstract

**Introduction:**

Truck drivers are exposed to long working hours, sleep deprivation, and social isolation, making them more vulnerable to mental disorders such as depression. However, population-based studies on mental health in this group remain scarce in Brazil.

**Objectives:**

To investigate the prevalence and factors associated with an elevated risk of depression among truck drivers.

**Methods:**

This cross-sectional study included 597 truck drivers and was conducted between April and July 2023 at fuel stations and seed factory parking lots in the municipalities of Formosa and Rio Verde, Goiás, Brazil. Participants completed a questionnaire assessing sociodemographic, occupational, behavioral, and psychosocial variables. Depression symptoms were assessed using the depression subscale of the DASS-21, with cutoff scores defined as follows: ≤13 indicating low risk and ≥14 indicating high risk of depression. Binary logistic regression was performed to identify predictors of elevated risk of depression.

**Results:**

The prevalence of an elevated risk of depression among participants was 13.6%. Drivers aged 60 years or older had higher odds of elevated depression risk, as did those with lower educational levels, particularly those who had not completed high school. Regarding occupational factors, working more than 12 hours per day was associated with a higher likelihood of depressive symptoms, as well as working irregular or night shifts. Furthermore, the number of kilometers driven daily was also a significant risk factor, with drivers covering more than 800 km per day showing the highest odds of elevated depression risk.

**Table 1.** Sociodemographic factors associated with elevated risk of depression among truck drivers Table 1. Long description.

Variable		
Age 41–60 (ref: ≤40 years)	1.08 (0.63 – 1.85)	0.787
Age >60 (ref: ≤40 years)	2.35 (1.12 – 4.94)	0.024
Less than high school (ref: high school or more)	2.02 (1.21 – 3.35)	0.007

**Table 2.** Occupational factors associated with elevated risk of depression among truck drivers Table 2. Long description.

Variable	Odds Ratio (95% CI)	p-value
9–12 working hours/day (ref: ≤8h)	1.72 (0.86 – 3.42)	0.123
>12 working hours/day (ref: ≤8h)	2.51 (1.16 – 5.42)	0.020
Morning, afternoon & night shift (ref: morning & afternoon)	1.98 (1.03 – 3.80)	0.040
Other shifts (ref: morning & afternoon)	2.92 (1.43 – 5.98)	0.003
501–800 km/day (ref: ≤500 km)	3.21 (1.64 – 6.28)	<0.001
>800 km/day (ref: ≤500 km)	9.27 (3.66 – 23.50)	<0.001

**Conclusions:**

The findings highlight that sociodemographic factors and working conditions significantly impact the mental health of truck drivers, reinforcing the need for public policies focused on the prevention of mental disorders and the promotion of well-being in this vulnerable population.

**Disclosure of Interest:**

None Declared

## Long descriptions

### Table 1. Long description

The table consists of three columns: Variable, Odds Ratio with 95 percent Confidence Interval, and p-value.

* Row 1: Age 41 to 60 (reference group: less than or equal to 40 years) shows an odds ratio of 1.08 (0.63 to 1.85) and a p-value of 0.787.

* Row 2: Age greater than 60 (reference group: less than or equal to 40 years) shows an odds ratio of 2.35 (1.12 to 4.94) and a p-value of 0.024.

* Row 3: Less than high school education (reference group: high school or more) shows an odds ratio of 2.02 (1.21 to 3.35) and a p-value of 0.007.

Navigate back to Table 1..

### Table 2. Long description

The table consists of three columns: Variable, Odds Ratio (95 percent C I), and p-value.

* 9 to 12 working hours per day (reference: less than or equal to 8 hours): Odds Ratio 1.72 (0.86 to 3.42), p-value 0.123.

* Greater than 12 working hours per day (reference: less than or equal to 8 hours): Odds Ratio 2.51 (1.16 to 5.42), p-value 0.020.

* Morning, afternoon and night shift (reference: morning and afternoon): Odds Ratio 1.98 (1.03 to 3.80), p-value 0.040.

* Other shifts (reference: morning and afternoon): Odds Ratio 2.92 (1.43 to 5.98), p-value 0.003.

* 501 to 800 km per day (reference: less than or equal to 500 km): Odds Ratio 3.21 (1.64 to 6.28), p-value less than 0.001.

* Greater than 800 km per day (reference: less than or equal to 500 km): Odds Ratio 9.27 (3.66 to 23.50), p-value less than 0.001.

Navigate back to Table 2..

